# An efficient approach for textual data classification using deep learning

**DOI:** 10.3389/fncom.2022.992296

**Published:** 2022-09-15

**Authors:** Abdullah Alqahtani, Habib Ullah Khan, Shtwai Alsubai, Mohemmed Sha, Ahmad Almadhor, Tayyab Iqbal, Sidra Abbas

**Affiliations:** ^1^College of Computer Engineering and Sciences, Prince Sattam Bin Abdulaziz University, Al-Kharj, Saudi Arabia; ^2^Department of Accounting and Information Systems, College of Business and Economics, Qatar University, Doha, Qatar; ^3^College of Computer and Information Sciences, Jouf University, Al-Kharj, Saudi Arabia; ^4^Department of Computer Science, FAST-NUCES, Islamabad, Pakistan; ^5^Department of Computer Science, COMSATS University, Islamabad, Pakistan

**Keywords:** text data, machine learning, deep learning, text classification, text categorization

## Abstract

Text categorization is an effective activity that can be accomplished using a variety of classification algorithms. In machine learning, the classifier is built by learning the features of categories from a set of preset training data. Similarly, deep learning offers enormous benefits for text classification since they execute highly accurately with lower-level engineering and processing. This paper employs machine and deep learning techniques to classify textual data. Textual data contains much useless information that must be pre-processed. We clean the data, impute missing values, and eliminate the repeated columns. Next, we employ machine learning algorithms: logistic regression, random forest, K-nearest neighbors (KNN), and deep learning algorithms: long short-term memory (LSTM), artificial neural network (ANN), and gated recurrent unit (GRU) for classification. Results reveal that LSTM achieves 92% accuracy outperforming all other model and baseline studies.

## 1. Introduction

Tags or categories are assigned to unstructured text using text classification (Bashir et al., 2021; Rao et al., 2021). Since it is so versatile, it is considered one of the most effective natural language processing methods because it can organize, categorize, and structure any text to deliver meaningful information and solve problems. Machine learning techniques such as natural language processing (NLP) enable computers to comprehend text much as humans do (Akram et al., 2022; Anwar et al., 2022). The ability to predict depression from text using an LSTM model with two hidden layers, substantial bias, and two dense layers of recurrent neural network (RNN) can help prevent mental illnesses and suicidal thoughts in people (Amanat et al., 2022). Text can be a very rich data source, but extracting data from it is difficult and time-consuming due to its unstructured nature. Natural language processing and machine learning, which fall under artificial intelligence, make sorting text data easier (Ibrahim et al., 2022).

Text classifiers can organize, arrange, and categorize almost any type of text, including documents, medical research, files, and text found on the internet (Amanat et al., 2022). Unstructured data accounts for over 80% of all data, with text being one of the most common categories. Because analyzing, comprehending, organizing, and sifting through text data is difficult and time-consuming due to its messy nature, most businesses do not exploit it to its full potential. Text classification is a technique in which we have to extract useful information from text (Bashir et al., 2022). This is where machine learning and text classification come into play. Companies can use text classifiers to quickly and cost-effectively arrange all relevant text types, including emails, legal documents, social media, surveys, and more (Abbasi et al., 2021, 2022; Hina et al., 2021a). Due to this technology, companies can save time studying text data, automate business processes, and make data-driven business choices. Many companies use text analysis tools to analyze the text. With text analysis tools, businesses can structure vast amounts of information, such as emails, chats, social media, support tickets, documents, and so on, in seconds instead of days. Therefore, we can dedicate more resources to critical tasks (Hina et al., 2021b; Rafat et al., 2022).

Robotics deep learning applications contribute to enormous issues that machine learning does not address (Koppu et al., 2020; Javed et al., 2022). Text classification can be done with super high accuracy using deep learning architectures since they require low-level engineering and computation. Two main deep learning architectures for text classification are convolutional neural networks (CNNs) and RNNs. We know that multiple algorithms are used in a progressive chain of events. Using different techniques simultaneously to process vast amounts of data is very similar to how the human brain works when making decisions. Deep learning algorithms require much more training data than traditional machine learning algorithms. Unlike traditional machine learning algorithms, they do not have a threshold for learning from training data. The performance of several machine learning and deep learning algorithms on the Titanic dataset is investigated in this paper.

Text classification is an essential advancement in the characterization of dialects. It may be the most effective method, based on different classification algorithms. This work is motivated by Ranjitha and Prasad (2020) in which different machine learning techniques like Hadoop map-reduce and naive Bayes classifiers are used to classify the data. It is observed that the performance evaluation of Gaussian naive Bayes is 72% and can be improved by using deep learning. The difficulty with Naive Bayes is that it assumes all features are independent and assigns 0 probability to categorical variables. This paper makes the below contributions.

- Propose an efficient approach for textual data classification using the Long-Short Term Memory algorithm.
- We employ various machine learning and deep learning algorithms to evaluate the classification performance and pick out the best classifier.
- Results reveal that LSTM performs well as it has multiple hidden layers, keeps useful information and discards useless information, and has a good hold on our dataset with a significant accuracy of 92%.

The rest of the article is organized into several sections as follows. Section 2 provides an overview of the related work. Then, in section 3, we provide the methodology of our approach and the algorithms we used in our work. Section 4 provides the results and discussion, and then finally, Section 5 concludes this paper and provides future work.

## 2. Related work

Using Big Data Hadoop approaches, such as Ayma et al. (2015), is a key direction in text classification. After the training data has been produced, the trained model is assigned to one of the class labels. As defined by Liu et al. (2013), the training stage is a more scientific step that often reflects limited data acquisition and significantly impacts the classification step. As a result, Hadoop Map Reduce has several limitations regarding text classification (Subramaniyaswamy et al., 2015) machine learning Technique (Ayma et al., 2015) is used to build and apply the Nave Bayes classifier. In-text document classification (Aghila and Vidhya, 2010), the Naive Bayes model is utilized, in which the document is considered an event, and the likelihood of nonoccurrence of terms is tested. Two types of models that the model could describe are Bernoulli and multinomial. The performance and improvement of the Naive Bayes classifier is a naive assumption for text classification matching the performance enhancement (Shathi et al., 2016).

Semi-NB approaches could be used to measure conditional independence while determining the likelihood of the model. In contrast to traditional processes, Bayesian strategies (Ozechowski, 2014) provide a unique and methodical method of combining primary data with information. Several approaches, such as the hidden Markov model, SVM (Tipping, 1999), and many others, could be reformulated using the Bayesian NB classifier. Recognizing various DDOS Attacks using a multinomial classifier for the model is demonstrated. The application layer DDOS attacks are explained, and a classifier-based technique is offered for malicious website visitors (Stevanovic et al., 2013), which stops and eradicates the attack based on a polynomial distributed model. Several studies have compared multinomial event models, and the Naive Bayes technique has been improved. Several smoothing techniques are employed when particular words in text documents are ignored or dropped. This work on smoothing approaches uses a multinomial model for brief texts. This linear approach has recently been discovered to be capable of overcoming the dimensionality curse and delivering real-time performance (Azarmehr et al., 2015).

Mechanical equipment technology and science have grown and developed dramatically during the last few decades. Spinning machinery is one of our current businesses' most crucial mechanical equipment. Such machines are used for long periods, often under complex and demanding conditions, resulting in component failure during operation, compromising worker safety, and financial loss. Because a large%age of rotating machine problems are directly related to these components, such as rolling bearings (Wang et al., 2012), maintaining the operating condition of rotating machinery components is critical. Rolling bearings can develop a variety of faults, including fractures in their various components, such as the outer ring, inner ring, rolling elements or cage (Yang et al., 2018) or chipping of the ball due to the working load and pressures caused by an unbalanced shaft, as well as fatigue failure. The patterns of vibration and noise generated by the gadget are affected and changed by these flaws.

A new hybrid model is developed, incorporating the benefits of linearity and nonlinearity and the impact of manual operations. The LSTM model combines the autoregressive integrated moving average (ARIMA) model (Fan et al., 2021). Production time series from three actual wells are analyzed to compare the ARIMA-LSTM and ARIMA-LSTM-DP hybrid models to the ARIMA, LSTM, and LSTM-DP models. The prediction accuracy is determined by evaluating four indices: root mean squared error (RMSE), mean absolute error (MAE), mean absolute%age error (MAPE), and similarity (Sim). The results show that the single ARIMA model outperforms the others in constant production drop curves. On the other hand, when it comes to evolving nonlinear data, the LSTM model outperforms the ARIMA model (Fan et al., 2021).

It is challenging to ensure that most datasets practitioners use to create commercial NLP applications are noise-free. While BERT has excelled at applying what it has learned to new use cases, it is yet unknown how BERT performs when configured for noisy text; however, if practitioners eliminate noise from their datasets while refining BERT to address use cases in industry (Srivastava et al., 2020). Additional modalities that the Transformer does not directly use are present in many real-world datasets. To integrate text and tabular (categorical and numerical) data with Transformers for downstream applications. The toolkit seamlessly interacts with Hugging Face's current API, including tokenization and the model hub, making it simple to download several pre-trained models (Gu and Budhkar, 2021). Many NLU tasks have benefited from data augmentation, especially those lacking data. Data Boost is a robust and user-friendly text augmentation framework that uses reinforcement learning-guided conditional generation to enhance data (Liu et al., 2020).

The issue with automated text anonymization must be solved before sensitive papers containing personal data can be shared securely. The main ideas behind text anonymization are discussed, along with a survey of current techniques (Lison et al., 2021). Anonymization techniques have been created in two disciplines, natural language processing and privacy-preserving data publishing, that hardly ever interact with one another. Due to intellectual property rights and profit-making considerations, private sector companies are typically unwilling to share their work. As a result, they hardly ever make their annotating frameworks and toolkits available to the general public (Buchanan and Ortega, 2022). Despite recently reported accomplishments of text-to-text models, it can be challenging to represent technical input and output for such models. The most obvious choice of output representations, where relations are given out in straightforward predicate logic statements, did not result in acceptable performance on the Clinical TempEval 2016 relation extraction test (Dligach et al., 2022). The best systems prompt one event at a time and produce findings on par with conventional pairwise temporal relation extraction systems. There exist a few studies on textual data classification on the titanic dataset. They lack performance. We propose a deep learning-based approach for textual data classification to overcome this limitation.

## 3. Methodology

**Figure 1:**
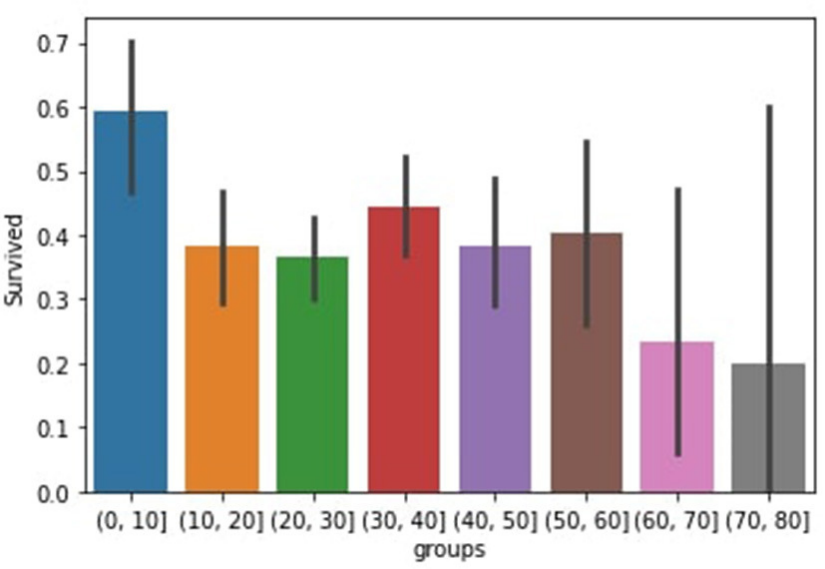
Survived in groups.

This research use *Titanic Disaster Dataset* that contains information about survivors, such as their gender, cabin, name, and age. First, we clear the data, then we impute missing information and values in The data, and then we select the categorical features means we pitch those valuable features and then divide them into training and testing Data after it has been imported. We calculate the likelihood probability and the Class label probability concerning the feature supplied in the training phase, and we estimate the probability for the given data using machine learning algorithms. We observe the number of persons who survived by sex by utilizing age groups as shown in Figure 1. In *Titanic Disaster Dataset* the number of males survival is very low than females by 2 and 7% this is because they want their children's and families to be safe, we extract this information from the data because we want to know how many individuals survived based on gender.

The proposed approach Algorithm 1 shows how our model works on this data, and we take data as input which contains information about survivors such as their gender, age, sex, and passenger's class. First, we clean the data, then impute missing information and values in the data using K-nearest neighbors (KNN) imputations. The next step was feature engineering in the proposed pipeline and discarding those features that were not useful. The next step was data normalization, i.e., Normalization reduces the effects of noise and outliers from the data and makes it computationally efficient. The last step in the pipeline was the model selection that generalized our dataset efficiently. For this purpose, we used numerous deep and machine learning models. We calculate the likelihood probability and the class Label Probability concerning the feature supplied in the training phase, and we estimate the probability that the persons from different groups survived or not.

**Algorithm 1:** Textual data analysis using LSTM.

**Input:** data ← Titanic Disaster Data
**Output:** survived or not survived
1: *V*←*LE*(*data*) {Label Encoding}
2: $\mu \leftarrow 1/m*\underset{i=1}{\sum }X^{\left(i\right)}$ {Normalizing data }
3: *X*←*X*−μ
4: $\sigma^{2}\leftarrow 1/m*\underset{i=1}{\sum }X^{\left(i\right)2}$
5: *X*/= σ^2^
6: *D*2←*np*.*array*(*Df*){Convergence of Matrix}
7: **for** *linrange*(1, *len*(*L*) **do** {Weight Initialization}
8: $W\left[l\right]\leftarrow rand\left(\left(m\times n\right)\right)*\sqrt{2/n\left[l-1\right]}$
9: **end for**
10: *V*←**MaxPooling**(*F*) {Conversion of Vector}
11: *lstm*←**LSTM**(*V*){LSTM layer}
12: *flstm*←**Hidden**(*lstm*){Hidden layer}
13: *PC*←**PredictClass**(*flstm*){Dense layer}
14: **for** *iinrange*(1, *len*(*PC*) **do**
15: **if** (*PC*[*i*] = *y*_*test*[*i*]) **then**
16: **return PC[i]**
17: **else**
18: **return y_test[i]**
19: **end if**
20: **end for**
21: **return** Output

**Figure 2:**
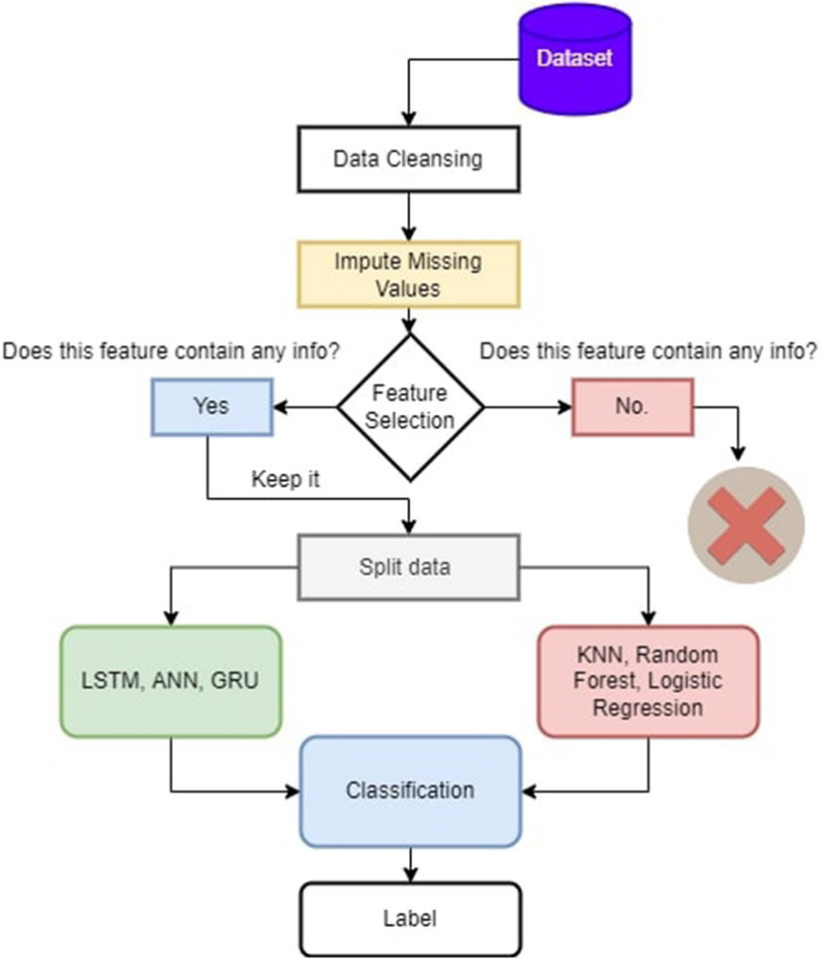
Methodology of the proposed approach.

Figure 2 presents the methodology of the paper. We impute missing values, and then we perform feature selection. The useful features like the number of people who survived in groups and sex are chosen, and then we split the data and perform deep learning and machine learning algorithms for classification to show how both the classifications work on titanic data. We also use GRU in RNNs since they contain a gating mechanism. The GRU is similar to a long-short-term memory (LSTM) with a forget gate but lacks an output gate. Hence it has fewer parameters. On the Titanic dataset, GRU performs, with up to 80% accuracy, because it only uses relevant data to create predictions. We also employ ANN, which employs learning algorithms that may change or train on their own as new data is received. Because we only use categorical features in ANN and eliminate irrelevant information, they are a very effective tool for non-linear statistical data modeling, as ANN exhibits up to 81% accuracy on this dataset. On the other hand, we use the dataset's K-nearest neighbor, random forest, and logistic regression. The logistic regression performs well as logistic regression predicts the categorical dependent variable's output using a collection of independent variables; it has a high degree of accuracy. So, logistic regression performs well in our scenario since our dataset has independent values, and we fill the missing values in separate classes, which is why machine learning techniques outperform base paper results.

## 4. Results and discussion

Table 1 provides the comparative analysis of the proposed approaches with other classifiers. LSTM contains a feature that allows it to remember the data sequence. It also has the advantage of working on the elimination of worthless information, and as we all know, text data contains much needless information, which the LSTM may remove to save calculation time and cost. So, the LSTM's ability to delete extraneous information while remembering the sequence of events makes it an excellent tool for text categorization. Next, We use GRUs since they feature a gating mechanism. The GRU is similar to an LSTM with a forget gate but lacks an output gate. Hence it has fewer parameters. GRU's performance on polyphonic music modeling, speech signal modeling, and natural language processing tasks was comparable to LSTM in some cases. On some smaller and less frequent datasets, GRUs have also performed better. We also apply ANN, which is a computational model that simulates the way nerve cells in the brain work. ANN employ learning algorithms that may make adjustments or learn on their own as new data is received. Because we only accept categorical features and eliminate irrelevant information in ANN, they are a very effective tool for non-linear statistical data modeling, as ANN exhibits up to 81% accuracy on this dataset. Machine learning algorithms on this data set include K-nearest neighbor, random forest, and logistic regression. Logistic regression predicts the output of the categorical dependent variable using a given set of independent variables with reasonable accuracy.

**Table 1:** Results of machine learning and deep learning techniques.

**Method**	**Accuracy(%)**
LSTM	92
GRU	80
Logistic regression	86
Random forest	82
ANN	81
KNN	77

**Figure 3:**
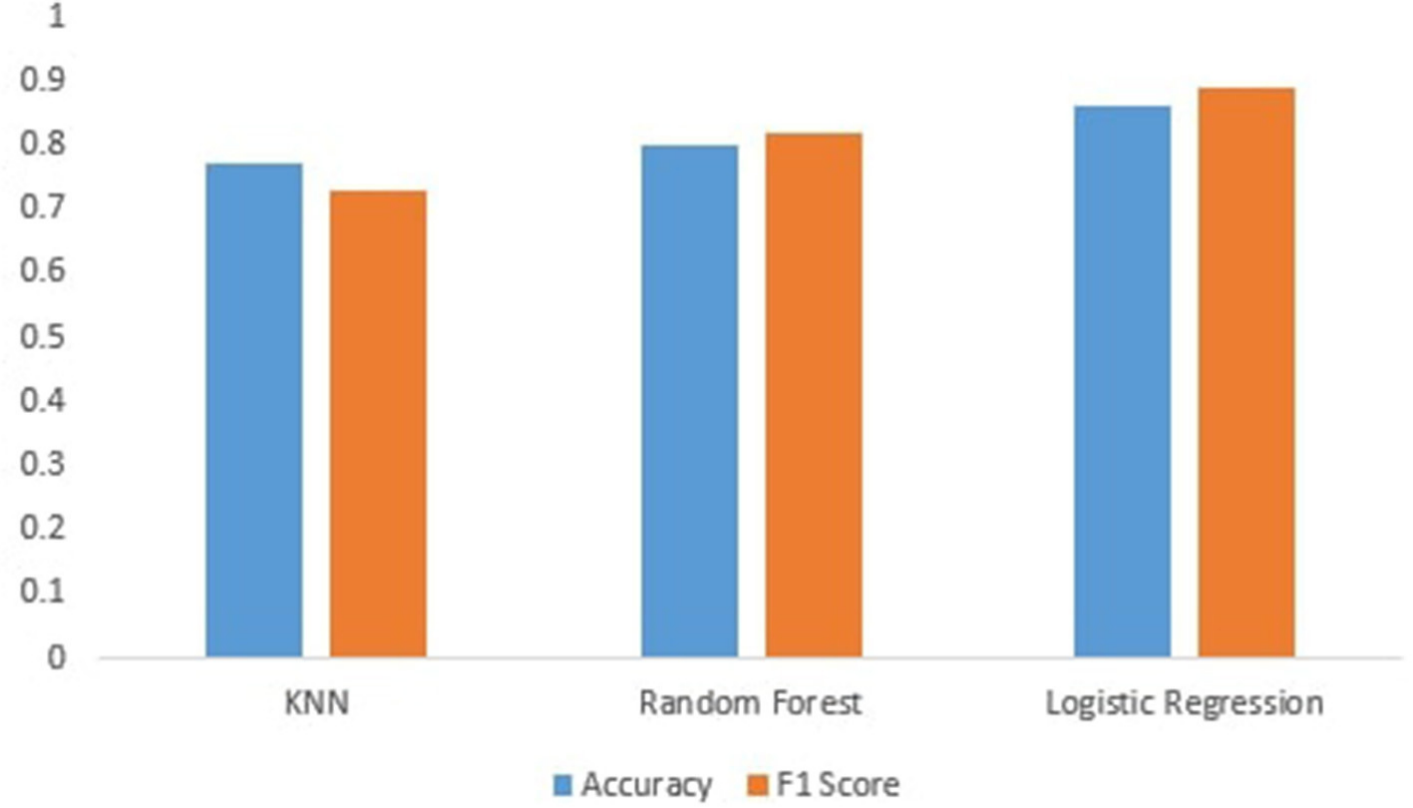
Accuracy and F1-score of machine learning models.

Figure 3 shows the performance of machine learning models. We can see that the machine learning models perform best after preprocessing data. It can be seen that KNN achieves an accuracy of 77%. This is because KNN is very sensitive to noisy data, and calculating the distance between each data point is very costly; and random forest achieves an accuracy of 82%, which is better than KNN, and for categorical features, it is found to be biased, and then, Logistic regression achieves high accuracy of 86% because logistic regression predicts the categorical dependent variable's output using a collection of independent variables. It has a high degree of accuracy. So, logistic regression performs well in our scenario since our dataset has independent values, and we fill the missing values in separate classes, which is why machine learning techniques outperform base paper results.

**Figure 4:**
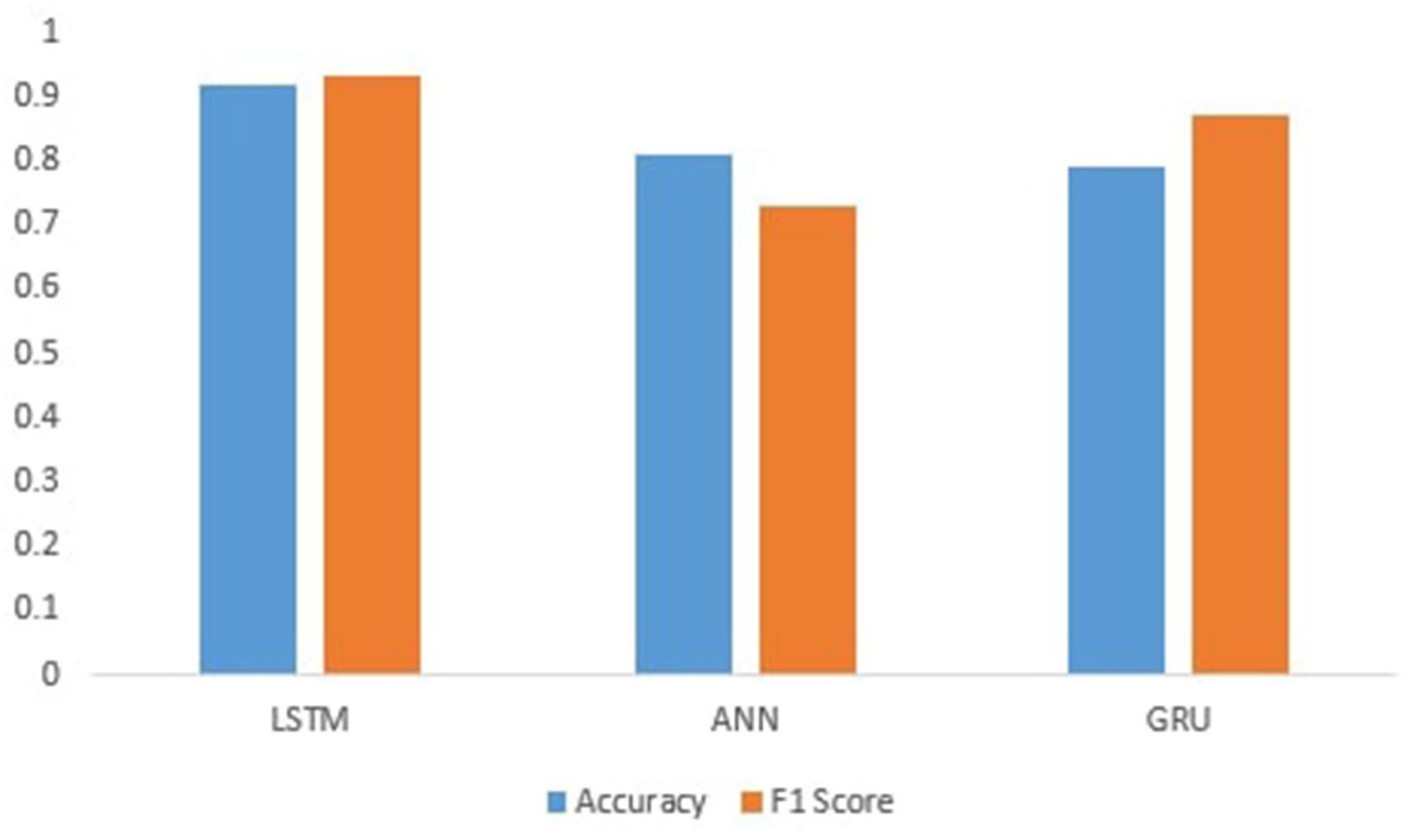
Accuracy and F1-score of deep learning models.

Figure 4 shows the performance of Deep learning models.ANN produces an accuracy of 81%. The GRU achieves an accuracy of 80%, which is less than LSTM because the separate concept of both the output at each time step and the cell memory is conceptualized in LSTM. The output and the hidden state for GRU are identical at every time step. This might help the LSTM learn some latent sequence properties that are not immediately related to elements in the sequence; that is why LSTM perform well on this data and shows the accuracy of 92% because it is a discriminative model and contains a feature that allows them to remember the data sequence. It also has the advantage of working on the elimination of worthless information, and as we all know, text data contains much needless information, which the LSTM may remove to save calculation time and cost. So, the LSTM's ability to delete extraneous information while remembering the sequence of events makes it an excellent tool for text categorization.

**Figure 5:**
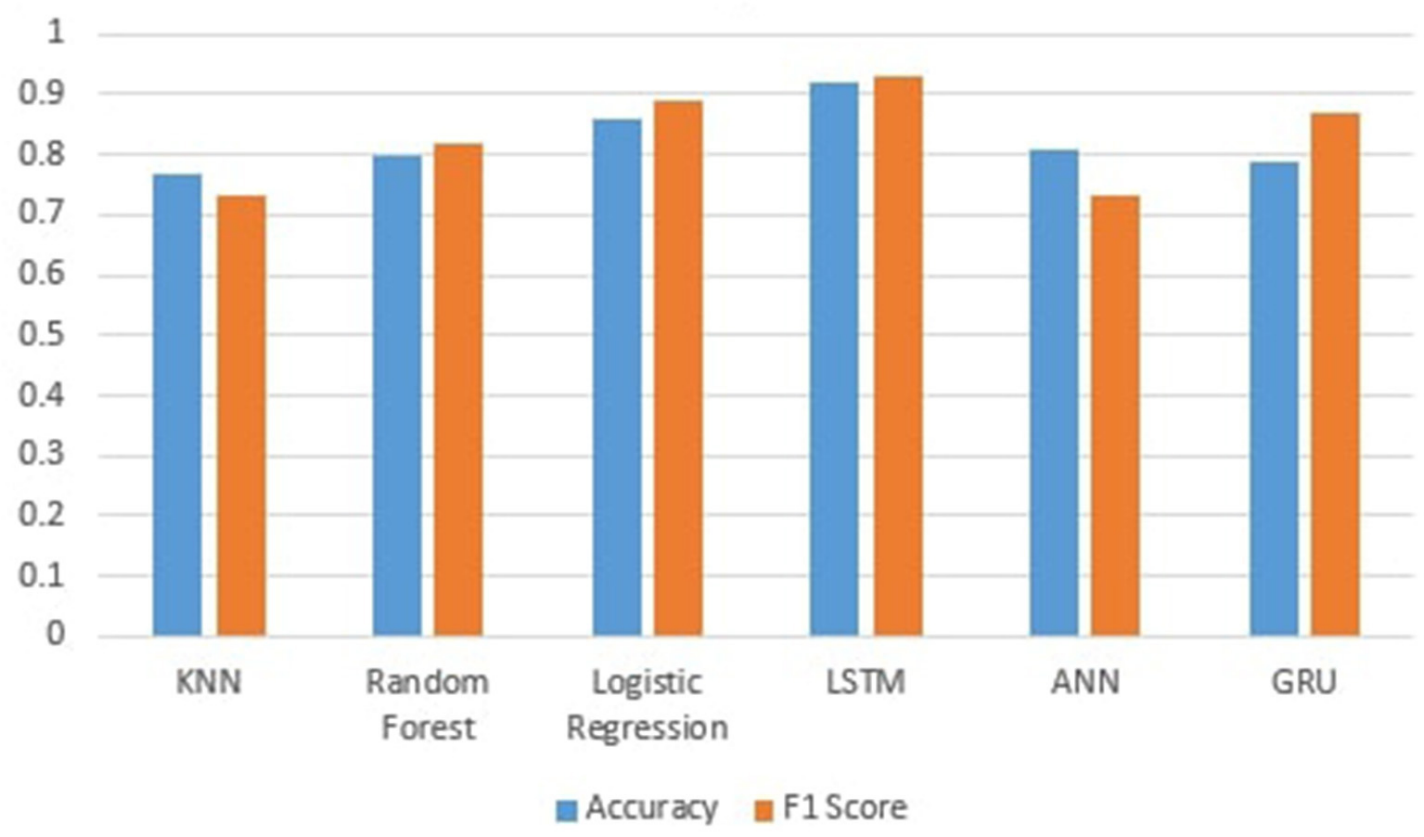
Comparison of machine learning and deep learning models.

Figure 5 shows the comparison of the machine and deep learning models in which LSTM performs significantly better than all other algorithms on this data, and the F1 score shows 93% shows the predictive performance of a model by combining precision and recall because it is the harmonic mean of precision and recall.

**Figure 6:**
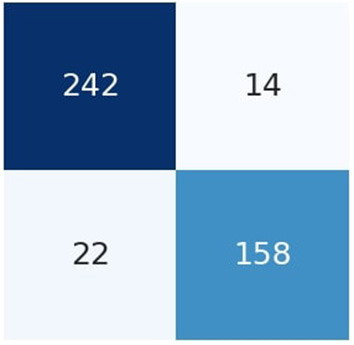
Confusion matrix of LSTM.

**Figure 7:**
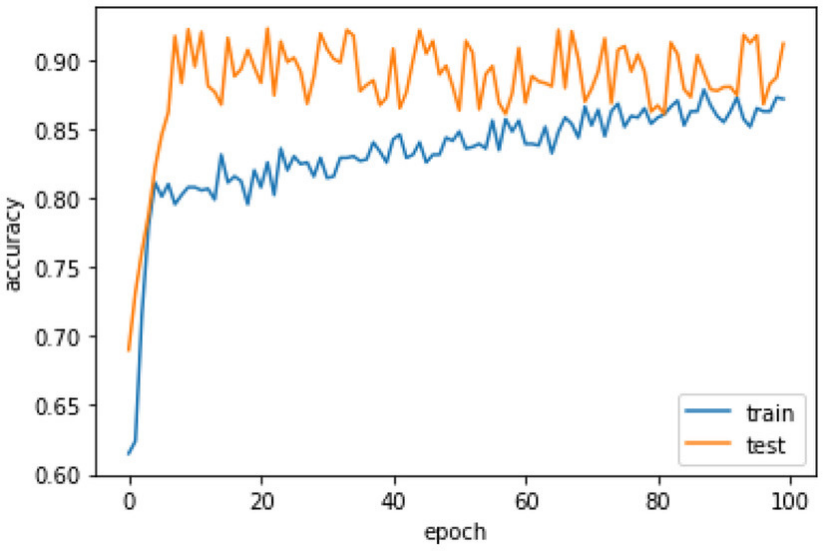
Accuracy curve of LSTM.

**Figure 8:**
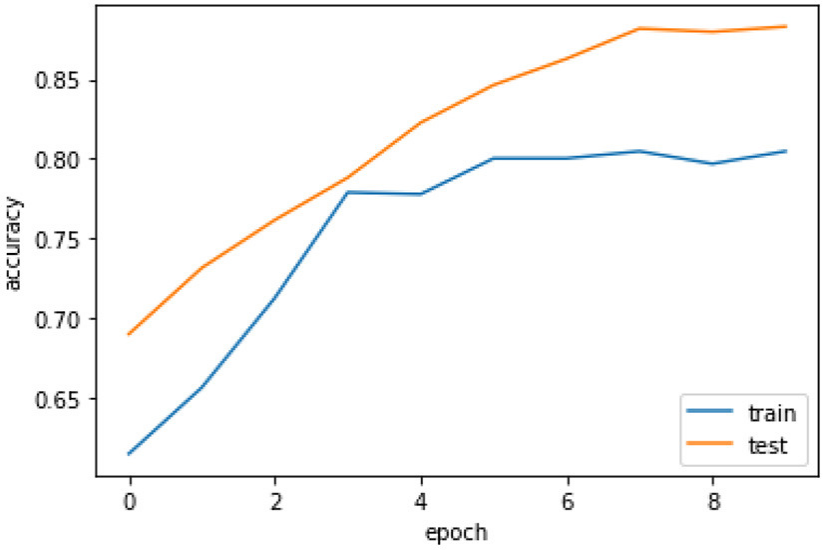
Accuracy curve of LSTM with early stopping.

Figure 6 the confusion matrix of LSTM depicts that 242 individuals are correctly classified as the survived people. Then, seeing the diagonal element TN, which is 158, means that people lose their lives, and then FP and FN represent the number of non-predicted values. The accuracy curve as shown in Figure 7 of LSTM shows that the proposed model when reaching the accuracy to 92% at epochs 10 and then it oscillates up to 100, so we use early stopping on our model the Figure 8 which clearly shows that how our model works on textual data.

**Figure 9:**
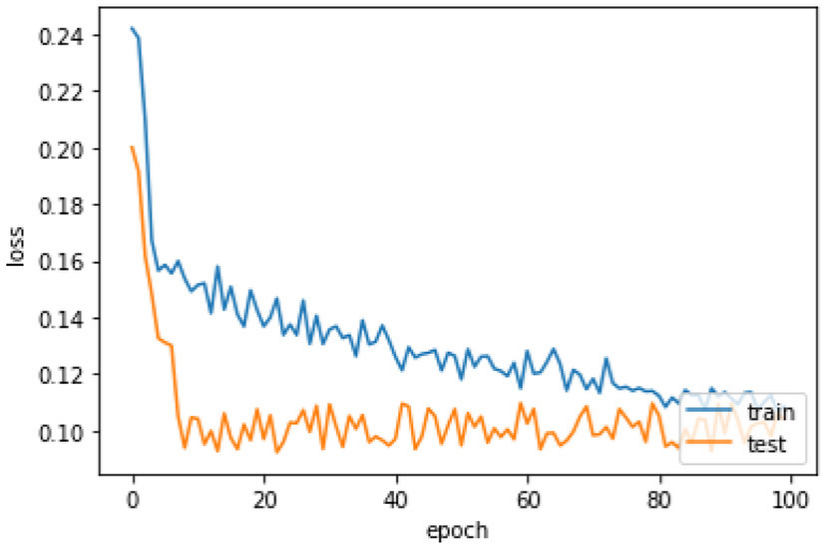
Loss curve of LSTM.

**Figure 10:**
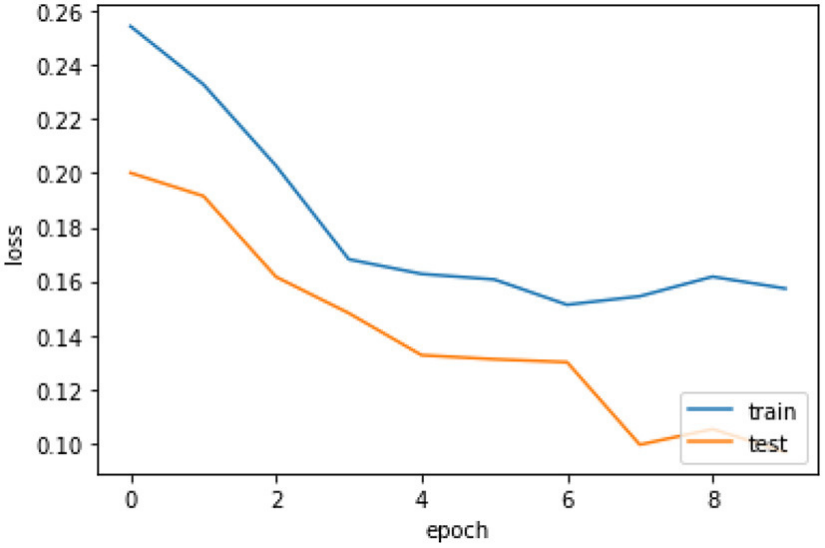
Loss curve of LSTM with early stopping.

The loss curve Figure 9 of LSTM shows that our model, when reaching to the loss to 16% at epochs 10 then oscillates up to 100 and also decreases the loss slowly, so we use early stopping on our model the Figure 10 which clearly shows the loss of accuracy on our data.

## 5. Conclusion and future outlook

There is an exponential increase in text data. As a result, it is not easy to manually sort through a large amount of data, so it is desired to discover feasible methods for quickly sorting through a large amount of data. After classification, the resulting data is called information, which is used to plan future business and industrial operations. This paper proposed an LSTM-based approach for textual data classification. We use several machine learning algorithms and deep learning algorithms for text classification. The LSTM remembers the order in which the data is presented for text data. It also has the advantage of working on the elimination of worthless information. LSTM's property of removing unnecessary information and remembering the sequence of the information makes it an excellent tool for text classification and other text-based tasks, as we improved the accuracy of the *Titanic Disaster Dataset* to 92%. In the future, we can undertake the categorization process utilizing numerous new deep learning algorithms, such as BERT. Most modern systems, like BERT, only take bidirectionality into account but also use a layered transformer and attention approaches that are highly parallelizable. It combines the most significant features of both recurrent and convolutional architectures and adds more, allowing it to perform even better.

## Data availability statement

The original contributions presented in the study are included in the article/supplementary material, further inquiries can be directed to the corresponding authors.

## Author contributions

All authors listed have made a substantial, direct, and intellectual contribution to the work and approved it for publication.

## Funding

The research is supported by Qatar National Library and Qatar university internal grant IRCC-2021-010.

## Conflict of interest

The authors declare that the research was conducted in the absence of any commercial or financial relationships that could be construed as a potential conflict of interest.

## Publisher's note

All claims expressed in this article are solely those of the authors and do not necessarily represent those of their affiliated organizations, or those of the publisher, the editors and the reviewers. Any product that may be evaluated in this article, or claim that may be made by its manufacturer, is not guaranteed or endorsed by the publisher.
